# Glycated haemoglobin (HbA1c) in mid-pregnancy and perinatal outcomes

**DOI:** 10.1093/ije/dyab270

**Published:** 2022-01-05

**Authors:** Ellen Ø Carlsen, Quaker Harmon, Maria C Magnus, Helle M Meltzer, Iris Erlund, Lars C Stene, Siri E Håberg, Allen J Wilcox

**Affiliations:** Centre for Fertility and Health, Norwegian Institute of Public Health, Oslo, Norway; Department of Community Medicine, Institute of Health and Society, University of Oslo, Oslo, Norway; Epidemiology Branch, National Institute of Environmental Health Sciences, Durham, NC, USA; Centre for Fertility and Health, Norwegian Institute of Public Health, Oslo, Norway; MRC Integrative Epidemiology Unit, University of Bristol, Bristol, UK; Population Health Sciences, Bristol Medical School, Bristol, UK; Division of Climate and Environment, Environment and Health, Norwegian Institute of Public Health, Oslo, Norway; Department of Government Services (Biomarkers Team), Finnish Institute for Health and Welfare, Helsinki, Finland; Department of Chronic Diseases and Ageing, Norwegian Institute of Public Health, Oslo, Norway; Centre for Fertility and Health, Norwegian Institute of Public Health, Oslo, Norway; Epidemiology Branch, National Institute of Environmental Health Sciences, Durham, NC, USA

**Keywords:** Birthweight, diabetes, glucose, HbA1c, preeclampsia, pregnancy, pregnancy complications, preterm, MoBa

## Abstract

**Background:**

Maternal diabetes is a well-known risk factor for pregnancy complications. Possible links between long-term maternal blood sugar in the normal range and pregnancy complications are less well described.

**Methods:**

We assayed glycated haemoglobin (HbA1c) in blood samples collected around the 18th week of pregnancy for 2937 singleton pregnancies in the Norwegian Mother, Father and Child Cohort Study (2000–09). Perinatal outcomes (gestational length, birthweight, birth length and head circumference, large-for-gestational age, small-for-gestational age, congenital malformations, preterm delivery and preeclampsia) were obtained from medical records. We tested associations using linear and log-binomial regression, adjusting for maternal age, body mass index (BMI) and smoking.

**Results:**

Size at birth increased modestly but linearly with HbA1c. Birthweight rose 0.10 standard deviations [95% confidence interval (CI): 0.03, 0.16], for each 5-mmol/mol unit increase in HbA1c, corresponding to about 40 g at 40 weeks of gestation. Large-for-gestational age rose 23% (95% CI: 1%, 50%) per five-unit increase. Other pregnancy complications increased in non-linear fashion, with strongest associations within the top quartile of HbA1c (>35 mmol/mol or >5.4%). Per unit HbA1c within the top quartile, preterm delivery increased by 14% (95% CI: 1%, 31%), preeclampsia increased by 20% (95% CI: 5%, 37%) and gestational duration decreased by 0.7 days (95% CI: −1.0, −0.3).

**Conclusions:**

Among women with no recorded diabetes, higher HbA1c levels at 18 gestational weeks were associated with important perinatal outcomes independent of mother’s age, smoking or BMI.

Key Messages

- High maternal glucose is a well-known risk factor for pregnancy complications.
- We find that normal variations in long-term maternal blood sugar, as measured by glycated haemoglobin (HbA1c), are related to pregnancy complications even among pregnant women without diabetes.
- Glycated haemoglobin (HbA1c) levels are linearly related to infant size at birth.
- Pregnant women with glycated haemoglobin (HbA1c) levels in the upper quartile (but still within the generally accepted normal range) are at increased risk of preterm delivery and preeclampsia.

## Introduction

Glycated haemoglobin (HbA1c) provides an integrated measure of blood glucose levels across the previous 90–120 days. HbA1c assays are standard for monitoring glycaemic control in patients with diabetes.^1^^,^^2^ Although monitoring blood glucose is particularly important during pregnancy (because hyperglycaemia is a recognized risk factor in pregnancy),^3–9^ HbA1c is not the preferred tool for pregnancy glucose monitoring. This is because increased haemoglobin turnover during pregnancy can slightly reduce HbA1c levels, and also because as an integrated measure of glucose, HbA1c may not adequately capture the short-term fluctuations regarded as important to diabetes management during pregnancy.^1^^,^^2^^,^^10–16^

For epidemiological purposes, however, the study of HbA1c in pregnancy has decided advantages. Not only is HbA1c simple to measure, but also the fact that it integrates maternal glucose levels across long periods of fetal development may make it a superior predictor of pregnancy complications. It is plausible that even within the normal range of maternal glucose, HbA1c may be linked with pregnancy outcomes. Until very recently, there has been little epidemiological research to explore this possibility.^2^^,^^17^ Only a handful of epidemiological studies have studied HbA1c in pregnancy in women without diabetes.^7^^,^^16–21^

Our purpose was to explore the normal variation in long-term maternal glucose levels measured at 18 weeks of gestation in pregnancies without diabetes, and to describe possible links with outcomes of pregnancy. We chose outcomes that have previously been associated with diabetes-related hyperglycaemia, including fetal growth, gestational duration, preeclampsia, preterm delivery and congenital malformations. We controlled for known risk factors such as age, body mass index (BMI) and smoking, which could potentially confound the associations with HbA1c.

## Methods

### Study design

Our study was based on a subsample of the Norwegian Mother, Father and Child Cohort Study (MoBa). MoBa recruited pregnant women across the nation between 1999 and 2008 at their routine ultrasound screening (at approximately 18 weeks).^22^ Participation rate was 41%. The cohort includes 95* *200 women and 114* *500 offspring (with some women contributing more than one pregnancy). In 2014, maternal HbA1c was measured in a random sample of 2979 singleton pregnancies with relatively complete data (Supplementary Methods S1, available as Supplementary data at *IJE* online).^23^ To avoid confounding by interventions for diabetes mellitus, we excluded 42 women (1.4%) registered with diabetes in the Medical Birth Registry^24^ (either pre-pregnancy, unspecified or gestational diabetes), leaving 2937 pregnancies for analysis. The prevalence of diabetes in this sample is slightly lower than the prevalence recorded among all pregnancies at birth during the same period (1.5–2.2%),^25^ reflecting the selection of relatively healthy women into MoBa.^23^

### Outcomes

The outcomes of interest were those that have been associated with frank hyperglycaemia: birthweight, birth length and head circumference, decreased gestational length and higher risk of preeclampsia, preterm birth and congenital malformations. Using unique personal identification numbers, pregnancies were linked to the Medical Birth Registry to obtain information on gestational age at delivery (days), birthweight (grams), birth length (centimetres), head circumference (centimetres), congenital malformations (any registered malformation) and preeclampsia [defined as a registration of preeclampsia, eclampsia or HELLP syndrome (haemolysis, elevated liver enzymes, low platelet count)]. Gestational age was based on routine ultrasound scan around week 18 (*n* = 2876) or on last menstrual period when ultrasound dating was missing (*n* = 51); 10 were missing both.

We calculated z-scores for birthweight, length and head circumference to allow standardization by sex, gestational age (whole weeks) and parity (0 or 1+). Singleton births in Norway between 1999 and 2017 were the reference population. Small-for-gestational age (SGA) was defined as birthweight less than the 10th percentile, and large-for-gestational age (LGA) as birthweight above the 90th percentile in the reference population. Preterm delivery was defined as delivery before 37 completed weeks.

### HbA1c assay

At a routine ultrasound visit around 18 gestational weeks, venous blood was collected and sent to the MoBa biobank for storage at −20ºC.^26^ We have no direct evidence comparing fresh samples with samples stored at −20ºC; HbA1c has been shown to be relatively stable in samples frozen at −80ºC.^27^ HbA1c was measured with an immunoturbidimetric method at the Biochemistry Laboratory, Forensic Toxicology Unit, Finnish Institute for Health and Welfare, Helsinki, Finland. The laboratory is accredited by the Finnish Accreditation Service (FINAS, Helsinki). HbA1c levels are reported as mmol/mol, and as corresponding values in % calculated with the formula: number in mmol/mol*0.0915) + 2.15.^28^^,^^29^ The samples were analyzed in two batches, December 2014–January 2015 and July–October 2015. The between-series precision expressed as coefficient of variation (CV) [mean ± standard deviation (SD)] was 2.0% ± 0.3 in the first and 1.8% ± 0.2 in the second batch. The between-series precision for the entire study (between-batch CV) was 1.9% ± 0.3. The laboratory took part in the HbA1c external quality assessment scheme organized by Lab quality (Helsinki, Finland). Trueness of the method was evaluated by using samples from the proficiency testing, with values assigned by the European Reference Laboratory for Glycohemoglobin. Systematic error (BIAS% ± SD) was 3.0% ± 1.4 and 4.1% ± 3.1, respectively, in the two batches (time periods). Further details of storage and measurements of HbA1c are provided in the Supplementary Methods S2 (available as Supplementary data at IJE online).

### Covariates

The Medical Birth Registry provided data on maternal age at delivery (whole years), parity, use of assisted reproductive technologies and child sex. MoBa cohort questionnaires provided information on self-reported height and pre-pregnancy weight [used to calculate body mass index (BMI, kg/m^2^)], smoking during pregnancy, educational level (less than high school, high school, college ≤4 years, college >4 years), weight gain during pregnancy and native language of the mother and her parents (Norwegian or other).

A challenge in an exploratory analysis of HbA1c during pregnancy is the limited knowledge about causal factors that determine it. Previous studies have varied widely in the selection of variables for adjustment.^7^^,^^17^ Given this uncertainty, we explored univariate and multivariate associations between HbA1c and a number of possible confounders, chosen for their plausible association with HbA1c and their known association with pregnancy outcomes. We saw few associations in our data; just three factors met criteria for confounding: maternal age, BMI and sustained smoking at time of recruitment (Supplementary Table S1 and Supplementary Figure S1, available as Supplementary data at *IJE* online). All our analyses adjust for these three factors.

We did not adjust for variables that are either related to prior pregnancy outcomes or associated only with HbA1c,^30^ as these variables cannot confound our associations. In the analysis of gestational age, we additionally adjusted for parity (primiparous, multiparous) in order to improve precision of the estimate. We do not necessarily assume a causal association between maternal HbA1c levels and pregnancy outcomes and we provide a directed acyclic graph (DAG) to describe our decisions with regard to adjustment for confounding factors (Supplementary Figure S2, available as Supplementary data at *IJE* online).

### Statistical analysis

Given the exploratory nature of this analysis, we chose a parsimonious multivariable analysis, adjusting for maternal age (continuous), BMI (continuous) and current smoking at week 18 (yes or no; smokers who quit before recruitment were included in the non-smoking category) and parity (0 versus 1+).

We used linear regression for continuous outcomes and log-binomial regression to estimate relative risks for binary outcomes. Initially, we explored relationships between HbA1c and outcomes in a flexible way, with a four-knot restricted cubic spline model. The relationship between HbA1c and standardized birthweight, birth length, head circumference and LGA were all approximately linear. There was no indication that restricted cubic splines improved the model fit over that with HbA1c as a linear continuous variable, and we used simple linear models in all further analyses (Supplementary Table S2, available as Supplementary data at *IJE* online). However, in our graphical displays of the regression analyses, we have used the four-knot restricted cubic spline model for transparency while using the simpler models for interpretable coefficients in the tables showing regression analyses.

The remaining outcomes—SGA, gestational age, preterm birth, preeclampsia and congenital malformations—appeared nonlinear in their association with HbA1c. Using likelihood ratio testing, we confirmed that a single-knot linear spline provided adequate model fit to all outcomes compared with a model using cubic splines with four knots (Supplementary Table S2). The location of the knot was determined by inspection of graphs with various knot locations. A knot between 34 and 35 mmol/mol (5.3% and 5.4%) adequately captured the inflection point for these outcomes, with the further advantage of dividing the study population into the top quartile (*n *= 710) and lower three quartiles (*n *= 2227). A recent paper^31^ identified impaired glucose metabolism in pregnancy at 5.7% or above (about 37 mmol/mol). We did not have sufficient power to examine associations at this extreme value.

Results are reported as follows. For outcomes showing a linear dose-response relationship with HbA1c, we estimated mean difference (for continuous outcomes) or relative risks (for binary outcomes) per five-unit increase in HbA1c (∼0.4% unit). For outcomes showing nonlinear dose-response relationships with HbA1c, we expressed estimates per one-unit increase in HbA1c. This more narrow interval was chosen to acknowledge the restricted range of HbA1c values above the knot (≥35 mmol/mol).

We explored the robustness of gestational age results by excluding preterm deliveries and pregnancies complicated by preeclampsia. In additional sensitivity analyses, we restricted to deliveries with a spontaneous onset of labour (as reported in the Medical Birth Registry) and separately conducted analyses stratified by parity. All analyses were performed using Stata (Statacorp, College Station, TX), version 15.0.

## Results

Characteristics of the 2937 mothers and newborns are shown in Table 1. Blood was sampled at a mean of 18.5 gestational weeks [standard deviation (SD) 1.3]. HbA1c values had a strongly Gaussian distribution (Supplementary Figure S3, available as Supplementary data at *IJE* online), with a mean of 32.7 mmol/mol (5.1%), an SD of 2.9 mmol/mol (0.26%) and a range of 22 to 47 mmol/mol (4.2–6.5%).

**Table 1:** Descriptive characteristics of the study population (*n* = 2937 singleton pregnancies). Norway, 2002–09

Characteristic	Total
Study sample/pregnancies, no.	2937
HbA1c (mmol/mol), mean (SD) [conversion to % Hb, (SD)]	32.7 (2.9) (5.1 (0.26))
Maternal age at delivery (years), mean (SD)	30.3 (4.2)
Maternal pre-pregnancy BMI (kg/m^2^), mean (SD)	23.9 (3.9)
Missing information on BMI, no. (%)	46 (1.6)
Primipara, no. (%)	1505 (51)
Smokers at time of recruitment to MoBa (daily or occasional), no. (%)	176 (6)
Quit smoking before recruitment (daily or occasional), no. (%)	361 (12)
Missing information on smoking, no. (%)	7 (0.2)
Gestational age at blood sampling (weeks), mean (SD)	18.5 (1.3)
Missing information on time of blood sampling, no. (%)	12 (0.4)
Gestational age at birth (weeks), mean (SD)	40.1 (1.4)
Missing information on gestational age, no. (%)	10 (0.3)
Preterm births, no. (%)	87 (3.0)
Preeclampsia, no. (%)	87 (3.0)
Any congenital anomaly, no. (%)	141 (4.8)
Birth length (cm), mean (SD)	50.5 (2.1)
Missing information on length, no. (%)	1 (0.03)
Head circumference (cm), mean (SD)	35.4 (1.6)
Missing information on head circumference, no. (%)	31 (1.1)
Birthweight (grams), mean (SD)	3653 (505)
Large-for-gestational age, no. (%)	369 (12.6)
Small-for-gestational age, no. (%)	188 (6.4)

Large-for-gestational age was defined as birthweight >90th percentile for gestational age in weeks, sex and parity (0 and 1+). Small-for-gestational age was defined as birthweight <10th percentile for the same parameters.

HbA1c, glycated haemoglobin; SD, standard deviation; BMI, body mass index; MoBa, the Norwegian Mother, Father and Child Cohort Study.

### Fetal growth

**Figure 1:**
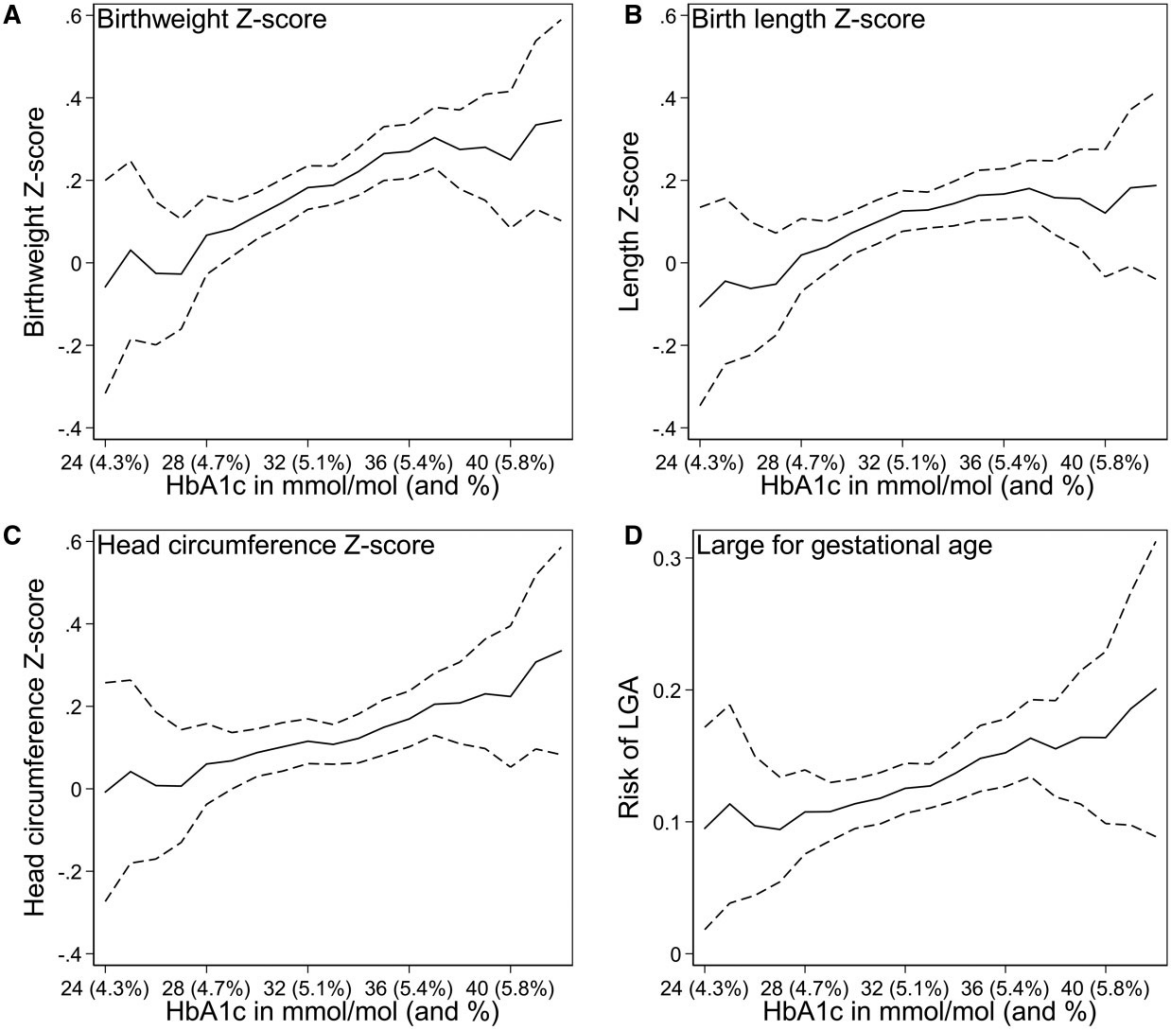
Birth size in relation to 18-week glycated haemoglobin (HbA1c) levels among up to 2874 singleton pregnancies, Norway, 2002–09. Panel A) Predicted birthweight z-score for HbA1c level. Panel B) Predicted birth length z-score for HbA1c level. Panel C) Predicted head circumference z-score for HbA1c level. Panel D) Predicted risk of large-for-gestational age (>90th percentile birthweight for sex, parity and gestational age in weeks) compared with normal-for-gestational age (10–90th percentiles). Solid lines show the predicted values, with dashed lines showing the 95% confidence intervals. Z-scores standardized for gestational age in weeks, parity and sex. All predicted outcomes are adjusted for body mass index (BMI) (set to mean), maternal age (set to mean) and smoking (set to non-smokers). The predictions are modelled using restricted cubic splines for HbA1c with four knots (placed at 28, 32, 34 and 38 mmol/mol). HbA1c levels were grouped at the extremes, with 42+ mmol/mol (6%) as the highest value (16 observations) and 24 mmol/mol (4.3%) as the lowest value (five observations).

Infant size, expressed in z-scores, increased linearly with higher HbA1c (Figure 1). Adjusted birthweight z-score increased 0.10 SD per 5-mmol/mol unit (∼0.4% unit) increase in HbA1c [95% confidence interval (CI): 0.03, 0.16, *P* for trend = 0.003] (Table 2). This is equivalent to about a 40-g increase at 40 weeks of gestation. Smaller increases were also present for head circumference (0.05 SD per five units of HbA1c; 95% CI: −0.01 to 0.12) and length (0.05 SD; 95% CI: −0.01 to 0.11) (Figure 1, Table 2). The relative risk (RR) of delivering an LGA baby increased 23% per five-unit increase in HbA1c (95% CI: 1%, 50%) (Figure 1, Table 2). The risk of SGA was elevated at low levels of HbA1c and then again at higher levels (Table 2; and Supplementary Figure S4, available as Supplementary data at *IJE* online). Since SGA babies may comprise both normal and pathologically small infants,^32^ our finding could suggest normal small growth at low levels of maternal blood glucose and abnormal growth restriction at high levels, although no firm interpretation is possible given the weak pattern.

**Table 2:** Linear and single-knot spline regression analyses for glycated haemoglobin (HbA1c) measured at 18 gestational weeks and multiple perinatal outcomes. Singleton pregnancies, Norway, 2002–09

Birth outcome^a^	HbA1c continuous, estimate per 5 units (95% CI)	HbA1c level ≤34 mmol/mol,^b^ estimate per unit (95% CI)	HbA1c level ≥35 mmol/mol,^b^ estimate per unit (95% CI)	Number of observations for each model, no. (% of study sample)
Birthweight (z-score)	0.10 (0.03, 0.16) *P* = 0.003	NA	NA	2874 (97.9)
Length (z-score)	0.05 (−0.01, 0.11) *P* = 0.09	NA	NA	2873 (97.8)
Head circumference (z-score)	0.05 (−0.01, 0.12) *P* = 0.10	NA	NA	2843 (96.8)
Large-for-gestational age^c^	1.23 (1.01, 1.50) *P *= 0.04	NA	NA	2874 (97.9)
Gestational age (days)	−0.81 (−1.47, −0.16) *P *= 0.02	0.07 (−0.12, 0.26) *P* = 0.49	−0.66 (−0.99, −0.33) *P* < 0.001	2874 (97.9)
Small-for-gestational age^c^	0.93 (0.72, 1.22) *P* = 0.62	0.95 (0.88, 1.02) *P* = 0.17	1.07 (0.95, 1.21) *P* = 0.27	2874 (97.9)
Preterm birth^c^	1.36 (0.95, 1.95) *P* = 0.09	1.01 (0.90, 1.13) *P* = 0.86	1.14 (1.00, 1.31) *P *= 0.05	2874 (97.9)
Preeclampsia^c^	1.07 (0.74, 1.54) *P* = 0.73	0.92 (0.83, 1.02) *P* = 0.12	1.20 (1.05, 1.37) *P* = 0.007	2884 (98.2)
Any congenital malformation^c^	0.93 (0.69, 1.24) *P* = 0.61	0.94 (0.86, 1.01) *P* = 0.10	1.10 (0.97, 1.25) *P* = 0.13	2884 (98.2)

a Adjusted for: maternal age (whole years), maternal pre-pregnancy body mass index (BMI) (kg/m^2^), smoking in pregnancy (yes versus no). Also adjusted for parity (0 versus 1+) for gestational age. Z-scores (birthweight, length, head circumference) and small- and large-for-gestational age standardized for gestational age in whole weeks, parity (0 vs 1+) and sex. Large-for-gestational age was defined as >90th percentile, and small-for-gestational age as <10th percentile.

b Showing the single-knot linear spline regression coefficients for the linear slope of HbA1c level up to and including 34 mmol/mol, and from 35 mmol/mol and up, respectively.

c Estimated risk ratio from multinomial regression model assessing risk of small-for-gestational age and large-for-gestational age compared with normal-for-gestational age. Estimated relative risk from binomial regression models for outcomes preterm birth, preeclampsia and any congenital malformation.

### Gestational age at birth

**Figure 2:**
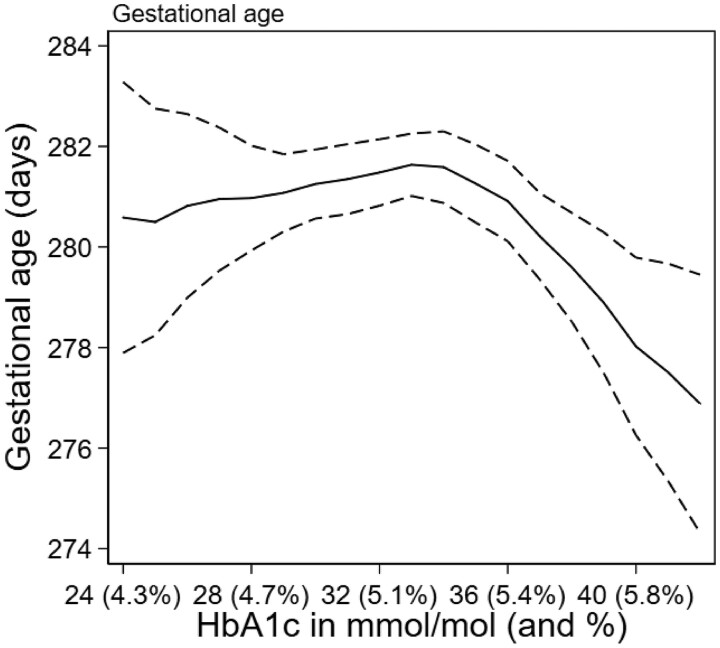
Gestational age at birth in relation to 18-week glycated haemoglobin (HbA1c) levels among 2874 singleton pregnancies, Norway, 2002–09. Predicted gestational age for each value of HbA1c (solid line), with 95% confidence intervals (dashed lines). The predictions were made using a linear regression model with restricted cubic splines for HbA1c with four knots (placed at 28, 32, 34 and 38 mmol/mol). Predictions were estimated using the adjusted models with the following covariates: maternal age (set to mean), body mass index (BMI) (set to mean), smoking (set to non-smokers) and parity (set to primipara). Note that HbA1c levels were grouped at the extremes, with 42+ mmol/mol (6%) as the highest value (16 observations) and 24 mmol/mol (4.3%) as the lowest value (five observations).

In a simple linear analysis, there was an association between HbA1c levels and decreasing gestational age (−0.8 days per five units of HbA1c; 95% CI: −1.5, −0.2). Model fit for this variable was improved with a spline model. The spline model suggests a weak association between HbA1c and gestational duration in the lower three quartiles of HbA1c but a marked decline within the top quartile (−0.7 days per one unit of HbA1c; 95% CI: −1.0, −0.3) (Table 2, Figure 2). Within the top quartile, this decline corresponds to a decrease of 3.3 days per five-unit increase in HbA1c (95% CI: −5.0, −1.7). The shortening of pregnancy within the upper quartile was robust in sensitivity analyses, including stratified analyses of births without preterm deliveries or preeclampsia (Supplementary Figure S5 and Supplementary Table S3, available as Supplementary data at *IJE* online).

### Preeclampsia, preterm birth and congenital malformations

**Figure 3:**
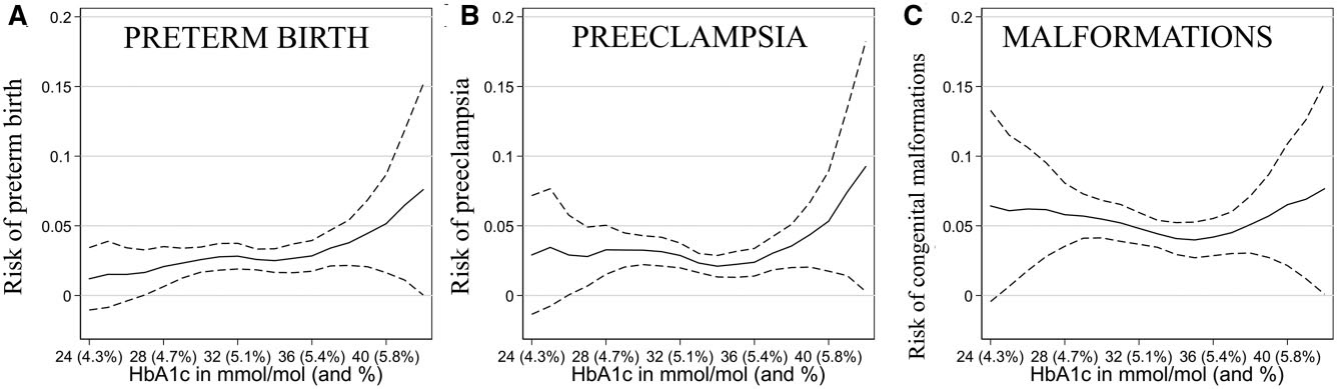
Pregnancy outcomes in relation to 18-week glycated haemoglobin (HbA1c) among up to 2874 singleton pregnancies, Norway, 2002–09. Panel A) Predicted risk of preterm birth. Panel B) Predicted risk of preeclampsia. Panel C) Predicted risk of any congenital malformation. Solid lines show the predicted values, with dashed lines showing the 95% confidence intervals. The predictions were made using a binomial regression model with restricted cubic splines for HbA1c with four knots (placed at 28, 32, 34 and 38 mmol/mol). Adjusted for maternal age (set to mean), body mass index (BMI) (set to mean), and smoking (set to non-smokers). Note that HbA1c levels were grouped at the extremes, with 42+ mmol/mol (6%) as the highest value (16 observations) and 24 mmol/mol (4.3%) as the lowest value (five observations).

The risks of preterm delivery and preeclampsia were both increased within the highest quartile of HbA1c. Preeclampsia increased 20% per unit increase of HbA1c (95% CI: 5%, 37%) and preterm delivery increased 14% per unit increase (95% CI: 0%, 31%) (Table 2, Figure 3). There was the suggestion of a slight increase in risk of congenital malformations in the upper quartile of HbA1c (RR 1.10; 95% CI: 0.97, 1.25) (Table 2, Figure 3).

## Discussion

In this population sample, maternal HbA1c at 18 gestational weeks was linearly associated with infant size at birth (weight, length, head circumference and LGA) independent of the mother’s BMI, age or smoking. Within the highest quartile of HbA1c (35 mmol/mol or greater), increasing HbA1c was related to shorter pregnancy duration and an increased risk of preeclampsia and preterm delivery. These observations were despite the fact that women with a diagnosis of diabetes had been excluded, and HbA1c values were in a range considered as normal.

### Previous literature

The types of adverse outcomes we observed are the same as (but less severe than) outcomes in pregnancies with gestational diabetes.^4^^,^^9^^,^^33^^,^^34^ Although there are few epidemiological studies on HbA1c in pregnant women without diabetes, the past studies generally support our results. The only large study of HbA1c very early in pregnancy (around 7 gestational weeks) found an increased risk of LGA, preterm delivery, preeclampsia and major congenital malformations among women in the extreme upper 3rd percentile of HbA1c concentrations.^7^ One previous study measured HbA1c late in pregnancy, around week 28. The study was designed primarily to compare HbA1c with other glucose measures, but the authors reported associations of HbA1c as a continuous measure with birthweight, preterm delivery and preeclampsia.^20^ Two recent studies have assessed HbA1c and pregnancy outcomes. Chen *et al*.^21^ considered the predictive power of HbA1c in the high range of normal (5.7%–6.4%, or approximately 38–47 mmol/mol) in early pregnancy, although they did not exclude pregnancies that developed gestational diabetes mellitus (GDM). Bi *et al*.^17^ assessed HbA1c continuously across quintiles, and addressed three of the outcomes in our analysis (birthweight, LGA and preterm delivery).

### Strengths and limitations

Our study participants came from a national pregnancy cohort, with the inevitable selection that occurs in volunteer studies. The impact of selection in this cohort has been explored for a few key aetiological associations, with only minor bias apparent.^35^ Further selection may have occurred because women with relatively complete data were chosen for HbA1c assay. However, our study was presumably less selective than studies that required women to submit to an oral glucose tolerance test.

We did not have information on maternal ethnicity, which has been a confounder in studies outside Norway.^2^^,^^36^ Our surrogate measure (native tongue other than Norwegian for the woman or her parents) was unrelated to HbA1c.

Our z-score measures of fetal size included gestational age as an adjustment variable. Adjusting for this mediating variable raises the theoretical possibility of collider stratification bias.^35^ However, not to adjust would raise the practical problem of failing to account for reduced birth size due to the shortened pregnancies associated with high HbA1c.

HbA1c can be affected by levels of blood haemoglobin, and we lacked information on this variable. Pathologies of haemoglobin are relatively rare. The anaemia associated with pregnancy could be a potential confounding factor, although this is expected to be relatively minor.^16^

Our analysis of congenital malformations was limited by lack of data on specific defects. It is possible that the weak trend we observed towards increased total malformations in the upper quartile of HbA1c reflects stronger increases of specific defects that have been associated with frank diabetes (e.g. cardiac defects).^37^ More detailed studies can test this hypothesis.

Information on diagnosis of preeclampsia was obtained from the Medical Birth Registry of Norway. The validity of this diagnosis has been described, indicating a high specificity but lower sensitivity, particularly among milder cases of preeclampsia.^38^ We cannot exclude the possibility that misclassification might have muted the association between HbA1c and preeclampsia in this study.

### Interpretation

The linear associations of HbA1c with infant size supports the hypothesis that fetal growth is a continuous function of maternal glucose levels, with no obvious threshold.^39^ Given that physiological changes of pregnancy can slightly reduce the apparent level of HbA1c at a given level of blood glucose, we did not rely on pre-specified criteria for high HbA1c. After excluding women with diabetes, we based our analysis on variation within the observed distribution of HbA1c, particularly within the upper quartile. Whereas this approach lacks the rigour of a pre-specified hypothesis, it is suitable for an exploratory description of a topic with limited prior history. Our nonlinear analyses are *post* *hoc*, and we give less weight to the exact magnitude of risk or the location of an HbA1c threshold than to the general observation that pregnancy complications apparently increase within the upper range of HbA1c in women without diabetes. Our results provide explicit hypotheses for testing in future studies.

It remains to be seen whether the increased risks we have identified with high-normal levels of HbA1c at week 18 can be mitigated by the dietary and exercise recommendations routinely provided later in pregnancy to women with gestational diabetes.^36^ It would also be of interest in future studies to explore whether these variations in normal HbA1c during pregnancy might be associated with outcomes in childhood, such as body size or neurodevelopment.

In conclusion, higher HbA1c levels in mid-pregnancy among women without diabetes were associated with larger infant size at birth and shorter pregnancy duration. Within the highest quartile of HbA1c, risk of preterm birth and preeclampsia also increased. Natural variation in long-term maternal glucose in women without diabetes, and independent of maternal BMI, smoking or age, may be a useful clinical predictor of pregnancy complications.

## Supplementary Data

Supplementary data are available at *IJE* online.

## Ethics approval

This project was approved by the Regional Committees for Medical and Health Research Ethics South East Norway (2014/434 and 2014/404). Data collection in MoBa was licensed by the Norwegian Data Protection Agency and approved by the Regional Committees for Medical and Health Research Ethics. The MoBa cohort is now regulated by the Norwegian Health Registry Act.

## Data availability

The individual-level data used in this study can only be given after approval by the Norwegian ethical committees approving that the applications are consistent with the consent provided. Access to the study dataset is available by application to the Norwegian Institute of Public Health using a form available on the English language portion of their website at [http://www.fhi.no/eway/].

## Funding

The Norwegian Mother, Father and Child Cohort Study is supported by the Norwegian Ministry of Health and Care Services and the Ministry of Education and Research. This work was funded in part by the Norwegian Research Council’s Centres of Excellence Funding Scheme (no. 262700). M.C.M works at the Medical Research Council Integrative Epidemiology Unit at the University of Bristol, which receives infrastructure funding from the UK MRC (MC_UU_00011/3 and MC_UU_00011/6). Support was also provided by the intramural programme of the National Institute of Environmental Health Sciences, National Institutes of Health, USA.

## Supplementary Material

dyab270_Supplementary_Data

## References

[1] Sacks DB , John WG. Interpretation of hemoglobin A1c values. JAMA 2014;311:2271–72. 24915255 10.1001/jama.2014.6342

[2] Rafat D , Ahmad J. HbA1c in pregnancy. Diabetes Metab Syndr 2012;6:59–64. 23014257 10.1016/j.dsx.2012.05.010

[3] Metzger BLL , Dyer AR, Trimble ER et al; for the HAPO Study Cooperative Research Group. Hyperglycemia and adverse pregnancy outcomes. N Engl J Med 2008;358:1991–2002. 18463375 10.1056/NEJMoa0707943

[4] Catalano PM , McIntyre HD, Cruickshank JK et al; HAPO Study Cooperative Research Group. The hyperglycaemia and adverse pregnancy outcome study: associations of GDM and obesity with pregnancy outcomes. Diabetes Care 2012;35:780–86. 22357187 10.2337/dc11-1790 PMC3308300

[5] Hapo Study Cooperative Research Group. Hyperglycaemia and Adverse Pregnancy Outcome (HAPO) Study: associations with neonatal anthropometrics. Diabetes 2009;58:453–59. 19011170 10.2337/db08-1112 PMC2628620

[6] Gomes D , von Kries R, Delius M et al Late-pregnancy dysglycemia in obese pregnancies after negative testing for gestational diabetes and risk of future childhood overweight: an interim analysis from a longitudinal mother-child cohort study. PLoS Med 2018;15:e1002681. 30372451 10.1371/journal.pmed.1002681 PMC6205663

[7] Hughes RC , Moore MP, Gullam JE, Mohamed K, Rowan J. An early pregnancy HbA1c >/=5.9% (41 mmol/mol) is optimal for detecting diabetes and identifies women at increased risk of adverse pregnancy outcomes. Diabetes Care 2014;37:2953–59. 25190675 10.2337/dc14-1312

[8] Ringholm L , Damm P, Mathiesen ER. Improving pregnancy outcomes in women with diabetes mellitus: modern management. Nat Rev Endocrinol 2019;15:406–16. 30948803 10.1038/s41574-019-0197-3

[9] World Health Organization. Diagnostic criteria and classification of hyperglycemia first detected in pregnancy: a World Health Organization Guideline. Diabetes Res Clin Prac 2014;103:341–63. 10.1016/j.diabres.2013.10.012 24847517

[10] Church D , Simmons D. More evidence of the problems of using HbA1c for diagnosing diabetes? The known knowns, the known unknowns and the unknown unknowns. J Intern Med 2014;276:171–73. 24443985 10.1111/joim.12200

[11] Lurie S , Mamet Y. Red blood cell survival and kinetics during pregnancy. Eur J Obst Gynecol Reprod Biol 2000;93:185–92. 11074141 10.1016/s0301-2115(00)00290-6

[12] Nielsen LR , Ekbom P, Damm P et al HbA1c levels are significantly lower in early and late pregnancy. Diabetes Care 2004;27:1200–01. 15111545 10.2337/diacare.27.5.1200

[13] Mosca A , Paleari R, Dalfrà MG et al Reference intervals for hemoglobin A1c in pregnant women: data from an Italian multicenter study. Clin Chem 2006;52:1138–43. 16601066 10.1373/clinchem.2005.064899

[14] O'Connor C , O'Shea PM, Owens LA et al Trimester-specific reference intervals for hemoglobin A1c (HbA1c) in pregnancy. Clin Chem Lab Med 2011;50:905–09. 22117781 10.1515/CCLM.2011.397

[15] O'Kane MJ , Lynch PL, Moles KW, Magee SE. Determination of a diabetes control and complications trial-aligned HbA(1c) reference range in pregnancy. Clin Chim Acta 2001;311:157–59. 11566175 10.1016/s0009-8981(01)00579-4

[16] Hughes RC , Rowan J, Florkowski CM. Is there a role for HbA1c in pregnancy? Curr Diab Rep 2016;16:5. 26739347 10.1007/s11892-015-0698-y

[17] Bi J , Ji C, Wu Y et al Association between maternal normal range HbA1c values and adverse birth outcomes. J Clin Endocrin Metabol 2020. doi: 10.1210/clinem/dgaa127. PMID: 32166332. 10.1210/clinem/dgaa127 32166332

[18] Li M , Hinkle SN, Grantz KL et al Glycaemic status during pregnancy and longitudinal measures of fetal growth in a multi-racial US population: a prospective cohort study. Lancet Diabetes Endocrinol 2020;8:292–300. 32135135 10.1016/S2213-8587(20)30024-3 PMC7676113

[19] Rasmussen KV , Nielsen KK, Pedersen ML. No association between early maternal HbA1c and offspring birthweight among women without pre-existing diabetes in Greenland. Int J Circum Health 2020;79:1702798. 10.1080/22423982.2019.1702798 PMC6913641 31825748

[20] Lowe LP , Metzger BE, Dyer AR et al; for the HAPO Study Cooperative Research Group. Hyperglycaemia and Adverse Pregnancy Outcome (HAPO) Study: associations of maternal A1c and glucose with pregnancy outcomes. Diabetes Care 2012;35:574–80. 22301123 10.2337/dc11-1687 PMC3322718

[21] Chen L , Pocobelli G, Yu O et al Early pregnancy hemoglobin A1c and pregnancy outcomes: a population-based study. Am J Perinatol 2019;36:1045–53. 30500961 10.1055/s-0038-1675619 PMC6612540

[22] Magnus P , Birke C, Vejrup K et al Cohort Profile Update: The Norwegian Mother and Child Cohort Study (MoBa). Int J Epidemiol 2016;45:382–88. 27063603 10.1093/ije/dyw029

[23] Caspersen IH , Thomsen C, Haug LS et al Patterns and dietary determinants of essential and toxic elements in blood measured in mid-pregnancy: the Norwegian Environmental Biobank. Sci Total Environ 2019;671:299–308. 30928759 10.1016/j.scitotenv.2019.03.291

[24] Irgens LM. Medical birth registry – an essential resource in perinatal medical research. Tidsskr Nor Laegeforen 2002;122:2546–69. 12522882

[25] Norwegian Institute of Public Health. *Medical Birth Registry – Statistics*. 2020. http://statistikkbank.fhi.no/mfr/ (6 October 2020, date last accessed)

[26] Paltiel L , Haugan A, Skjerden T et al The biobank of the Norwegian Mother and Child Cohort Study – present status. Nor J Epidemiol 2014. doi.org/10.5324/nje.v24i1

[27] Selvin E , Coresh J, Jordahl J, Boland L, Steffes MW. Stability of haemoglobin A1c (HbA1c) measurements from frozen whole blood samples stored for over a decade. Diabet Med 2005;22:1726–30. 16401319 10.1111/j.1464-5491.2005.01705.x

[28] Heinemann L , Freckmann G. Quality of HbA1c measurement in the practice: The German Perspective. J Diabetes Sci Technol 2015;9:687–95. 25691655 10.1177/1932296815572254 PMC4604529

[29] *National Glycohemoglobin Standardization Program, Harmonizing Haemoglobin A1c Testing*. 2010. http://www.ngsp.org/index.asp (6 October 2010, date last accessed).

[30] Howards PP , Schisterman EF, Heagerty PJ. Potential confounding by exposure history and prior outcomes: an example from perinatal epidemiology. Epidemiology 2007;18:544–51. 17879426 10.1097/ede.0b013e31812001e6

[31] Bozkurt L , Göbl CS, Leitner K, Pacini G, Kautzky-Willer A. HbA1c during early pregnancy reflects beta-cell dysfunction in women developing GDM. BMJ Open Diab Res Care 2020;8:e001751. 10.1136/bmjdrc-2020-001751 PMC7607595 33132213

[32] Mandy GT. *Infants with Fetal (Intrauterine) Growth Restriction, UpToDate*. 2021. https://www.uptodate.com/contents/infants-with-fetal-intrauterine-growth-restriction?search=Mandy%20GT.%20Infants%20with%20fetal%20(intrauterine)%20growth%20restriction&source=search_result&selectedTitle=1~150&usage_type=default&display_rank=1 (2 September 2021, date last accessed).

[33] Metzger BE , Gabbe SG, Persson B et al; International Association of Diabetes Pregnancy Study Groups Consensus Panel. International Association of Diabetes and Pregnancy Study Groups recommendations on the diagnosis and classification of hyperglycaemia in pregnancy. Diabetes Care 2010;33:676–82. 20190296 10.2337/dc09-1848 PMC2827530

[34] Wendland EM , Torloni MR, Falavigna M et al Gestational diabetes and pregnancy outcomes - a systematic review of the World Health Organization (WHO) and the International Association of Diabetes in Pregnancy Study Groups (IADPSG) diagnostic criteria. BMC Pregnancy Childbirth 2012;12:23. 22462760 10.1186/1471-2393-12-23 PMC3352245

[35] Nilsen RM , Vollset SE, Gjessing HK et al Self-selection and bias in a large prospective pregnancy cohort in Norway. Paediatr Perinat Epidemiol 2009;23:597–608. 19840297 10.1111/j.1365-3016.2009.01062.x

[36] Crowther CA , Hiller JE, Moss JR, McPhee AJ, Jeffries WS, Robinson JS. Effect of treatment of gestational diabetes mellitus on pregnancy outcomes. N Engl J Med 2005;352:2477–86. 15951574 10.1056/NEJMoa042973

[37] Eidem I , Stene LC, Henriksen T et al Congenital anomalies in newborns of women with type 1 diabetes: nationwide population-based study in Norway, 1999-2004. Acta Obstet Gynecol Scand 2010;89:1403–11. 20929418 10.3109/00016349.2010.518594

[38] Klungsøyr K , Harmon QE, Skard LB et al Validity of pre-eclampsia registration in the Medical Birth Registry of Norway for women participating in the Norwegian Mother and Child Cohort Study, 1999-2010. Paediatr Perinat Epidemiol 2014;28:362–71. 25040774 10.1111/ppe.12138 PMC4167249

[39] Freinkel N. Banting Lecture 1980. Of pregnancy and progeny. Diabetes 1980;29:1023–35. 7002669 10.2337/diab.29.12.1023

