# Supplementary material for: Glycated haemoglobin (HbA1c) in mid-pregnancy and perinatal outcomes
Source: Int J Epidemiol. 2022 Jan 5;51(3):759–68. doi: 10.1093/ije/dyab270 (PMC9189945; doi:10.1093/ije/dyab270)
Supplement: dyab270_Supplementary_Data [file dyab270_supplementary_data.docx]

**Supplementary Material**

**Table of contents**

Methods S1: Details of data sources and linkages…………………………………….……...2

Methods S2: HbA1c measurements………….....…………………………………….……....3

Figure S1: DAG showing selection of variables as possible confounders ………..................4

Figure S2: Predicted HbA1c levels by maternal age and maternal BMI………….................. 5

Figure S3: Distribution of HbA1c levels for the study population without diabetes.................6

Figure S4: Predicted risk of small-for-gestational age infant by HbA1c levels.........................7

Figure S5: Sensitivity analyses gestational age..........................................................................8

Table S1: Associations between covariates and HbA1c levels..................................................9

Table S2: Likelihood ratio-testing of regression models..........................................................10

Table S3: Regression table of sensitivity analyses of gestational age......................................11

References ...............................................................................................................................12

**Supplementary Methods S1:** Details of data sources and linkages

*The Medical Birth Registry of Norway*

The Medical Birth Registry of Norway was established in 1967 as the first National birth registry in the world.^1^ It is mandatory for midwives or the obstetrician to complete a birth record for all deliveries after 12 gestational weeks. Information on the birth record includes maternal demographic characteristics, parity, pre-existing chronic diseases and pregnancy complications. Information on child characteristics include sex, birthweight, length at birth, head circumference and gestational age at birth. Each woman has a unique personal identification number (PIN) used to link the information of the Norwegian Mother, Father and Child Cohort study.

*The Norwegian Mother, Father and Child Cohort Study (MoBa)*

Questionnaires were administered to mothers at 15 and 30 gestational weeks during pregnancy and at regular intervals after birth throughout childhood. Data collection after age 7 is ongoing. The questionnaires gathered a broad range of information, including socio-economic factors (marital status, education and income), lifestyle factors (smoking, body-mass index etc.) health status (chronic and infectious diseases) and anthropometric measures (height and weight). We used data available in November 2015 (V.9 of the quality assured data files).

The Norwegian Mother, Father and Child Cohort Study is supported by the Norwegian Ministry of Health and Care Services and the Ministry of Education and Research. We are grateful to all the participating families in Norway who take part in this on-going cohort study.

*The present study is embedded within the first phase of the Norwegian Environmental Biobank, a sub-study of the Norwegian Mother, Father and Child Cohort study*

The Norwegian Environmental Biobank was given permission to draw biological material from the MoBa biobank, including blood samples drawn in week 17-18 of pregnancy.^2-5^ For that study, participants were selected based on availability of all biological samples from mid-pregnancy and at birth, and only women who had answered all questionnaires up until 3 years after giving birth were eligible. Only singleton pregnancies were included, and pregnancies with children with autism, suspected autism, or symptoms of severe language delay were excluded. A total of n=2999 pregnancies were included in the study.^5^ For our study exploring HbA1c in mid-pregnancy, we included all pregnancies within the Norwegian Environmental Biobank study with a valid measurement of HbA1c, leaving a study population of n=2979 singleton pregnancies. For our main analyses, we excluded women with any diagnosis of diabetes mellitus (Type 1, n=8; Type 2, n=6; Unspecified, n=1; Gestational, n=24; registration of diabetic medication during pregnancy, n=3) as registered in the Medical Birth Registry of Norway, leaving a main study population for analyses consisting of n=2937 singleton pregnancies.

**Supplementary Methods S2**: glycated haemoglobin (HbA1c) measurements

Samples were stored at -20 ºC until analysis, for a range of 5-12 years. Glycated haemoglobin (HbA1c) is relatively stable in frozen samples.^6,7^ HbA1c was measured with an immunoturbidimetric method using an Architect c8000 analyzer (Abbott Laboratories, Abbott Park, Illinois, USA) at the Biochemistry Laboratory, Forensic Toxicology Unit, Finnish Institute for Health and Welfare, Helsinki, Finland. The laboratory is accredited by the Finnish Accreditation Service (FINAS, Helsinki) and fulfills the requirements of the standard SFS-EN ISO/IEC 17025:2005. The scope of accreditation (T077) covers the HbA1C assay. The samples were analyzed in two batches, December 2014-January 2015 and July-October 2015. The between-series precision expressed as coefficient of variation (CV) [mean ± standard deviation (SD)] was 2.0% ±0.3 in the first and 1.8% ±0.2 in the second batch. The between-series precision for the entire study (between-batch CV) was 1.9 % ±0.3. The laboratory took part in the HbA1c external quality assessment scheme organized by Lab quality (Helsinki, Finland). Trueness of the method was evaluated by using samples from the proficiency testing, with values assigned by the European Reference Laboratory for Glycohemoglobin. Systematic error (BIAS% ±SD) was 3.0 % ±1.4 and 4.1 % ±3.1, respectively, during the time periods.

A method comparison between the immunoturbidimetric method and an enzymatic method by Abbott Laboratories was also conducted at the Biochemistry Laboratory.^8^ Samples obtained from 100 subjects of four different nationalities (Finnish, Somali, Russian and Kurdish, n=25 of each) were analyzed by both methods. Very good agreement was observed.^8^ The regression equation of the immunoturbidimetric versus enzymatic method was y = 0,988x - 1,026, R² = 0,983.

We did not have information about factors interfering with HbA1c measurements, such as hemoglobinopathies,^9-11^ which could have biased the results due to unmeasured confounding. However, considering the fact that the genetic variants of hemoglobin shown to affect the accuracy of the method used in our study i.e., the HbS and HbC traits,^9^ are very uncommon in the Norwegian population,^12^ we do not expect this to affect our results.

The use of HbA1c to diagnose diabetes in pregnancy is controversial. The World Health Organization (WHO) and the International Association of Diabetes and Pregnancy Study Groups (IADPSG) recommend that HbA1c equal to or greater than 48 mmol/mol (6.5%) can be used to diagnose overt diabetes mellitus in pregnancy.^13,14^ The American Diabetes Association and IADPSG advise against the use of HbA1c for diagnosis in the second and third trimesters of pregnancy.^15,16^ No women in our study had an HbA1c concentration ≥48 mmol/mol (6.5%) after excluding those with a registered diagnosis of diabetes.

**Supplementary Figure S1**: Directed acyclic graph showing the selection of variables as potential confounding variables (in box) and other non-confounding variables of the associations between glycated haemoglobin (HbA1c) and selected pregnancy outcomes.


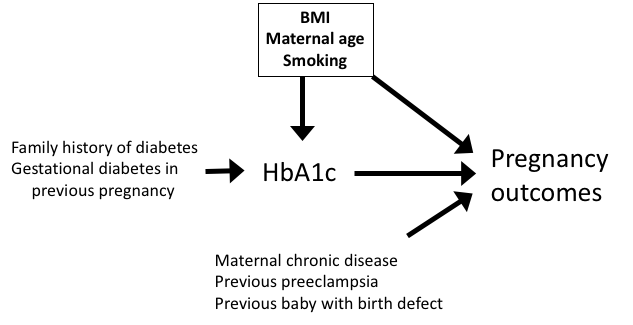


**Supplementary Figure S2**: Predicted levels of glycated haemoglobin (HbA1c) (mmol/mol) for maternal age and pre-pregnancy body mass index (BMI, kg/m^2^), Norway, 2002-2009, in n=2891 singleton pregnancies in mothers without diabetes mellitus, 98.4% of the study population.


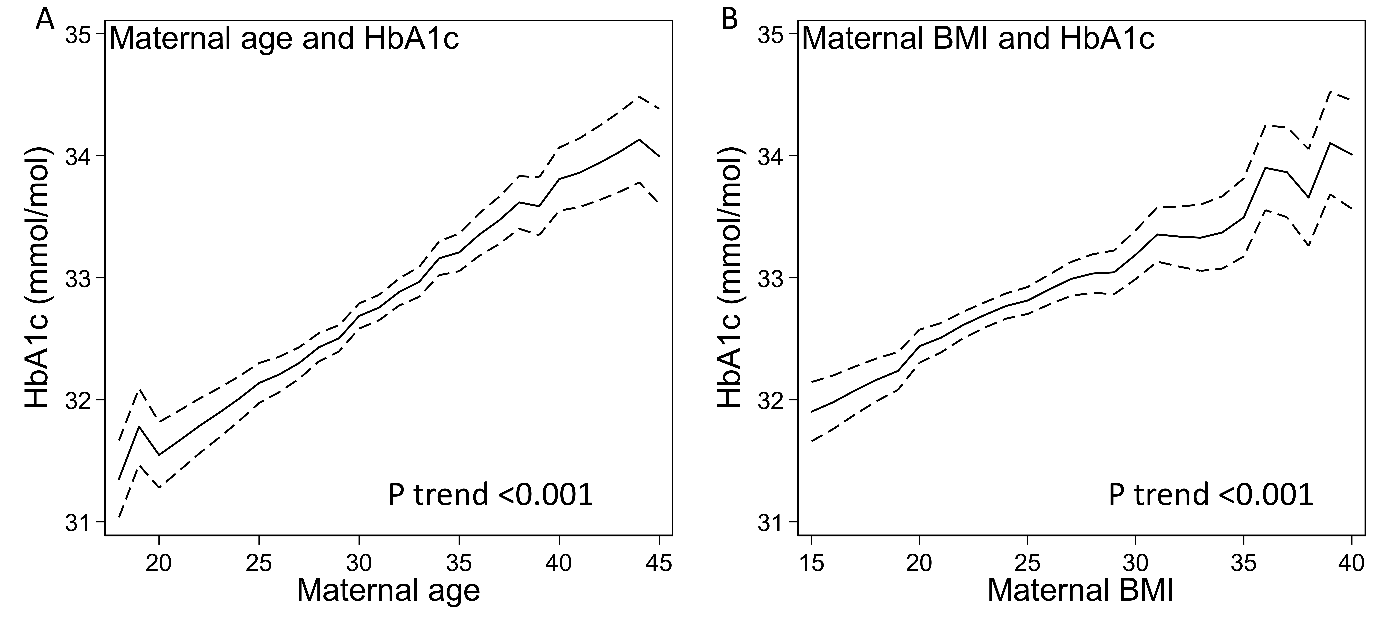


A) maternal age adjusted for maternal body mass index (BMI) (set to mean) and B) maternal pre-pregnancy BMI adjusted for maternal age (set to mean). Solid lines show predicted values; dashed lines show the 95% confidence intervals. Prediction based on a linear regression model with only the covariates HbA1c, maternal age and maternal BMI included. Excluded from the analyses if unavailable information on either of the covariates.

**Supplementary Figure S3**: Distribution of glycated haemoglobin (HbA1c) levels^a^ among 2937 women without registered diabetes mellitus at 18 weeks of pregnancy. Singleton pregnancies in Norway, 2002-2009.


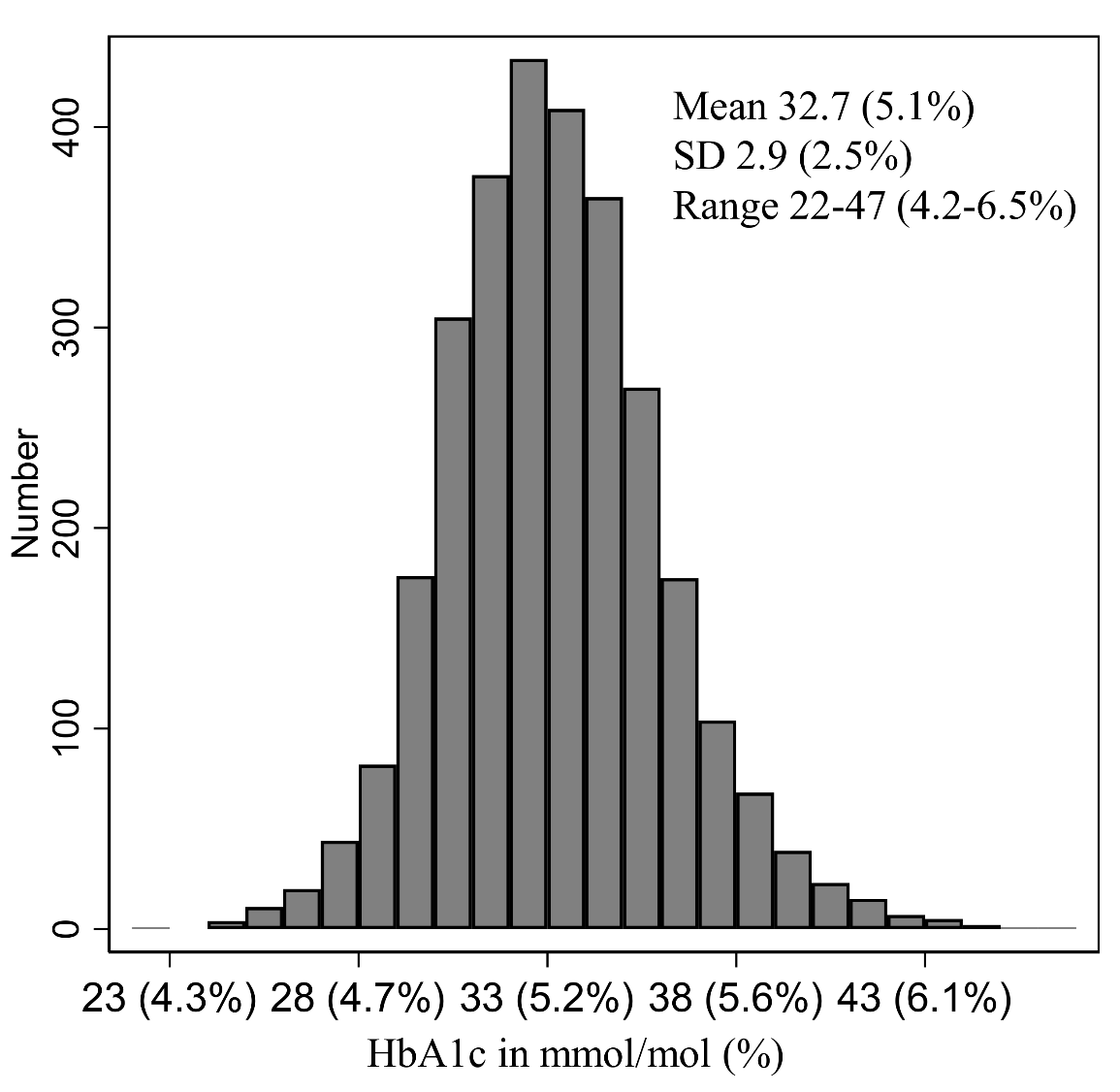


^a^ Results in parentheses show the corresponding HbA1c value in %-units, calculated using the formula “(*number in mmol/mol*0.0915)+2.15”.*

**Supplementary Figure S4**: Predicted risk^a^ of giving birth to a small-for-gestational age (SGA) infant in relation to 18-week glycated haemoglobin (HbA1c) levels. Solid line show the predicted values, and dashed lines show the 95% confidence intervals.

^a^ The predictions were made using a logistic regression model with restricted cubic splines for HbA1c with 4 knots (placed at 28, 31, 34 and 38 mmol/mol). Predictions were estimated using the adjusted models with the following covariates: maternal age (set to mean), body mass index (BMI) (set to mean) and smoking (set to non-smokers). Small-for-gestational age (SGA) was defined as a birthweight <10th percentile for gestational age in weeks, sex and parity (0 or 1+). The model compared odds of SGA versus normal for gestational age (a birthweight at the 10-90th percentiles for the given parameters).

Note that HbA1c levels were grouped at the extremes, with 42+ mmol/mol (6%) as the highest value (16 observations) and 24 mmol/mol (4.3%) as the lowest value (5 observations).

**Supplementary Figure S5**: Gestational age in relation to 18-week glycated haemoglobin (HbA1c) levels, sensitivity analyses. Panel A) removed preterm births and pregnancies with preeclampsia.^a^ Panel B) included only births with a reported spontaneous start of delivery. Panel C) included multiparous women only. Panel D) included primiparous women only. The graphs show the predicted gestational duration for each value of HbA1c (solid line), with 95% confidence intervals (between dashed lines). ^b^


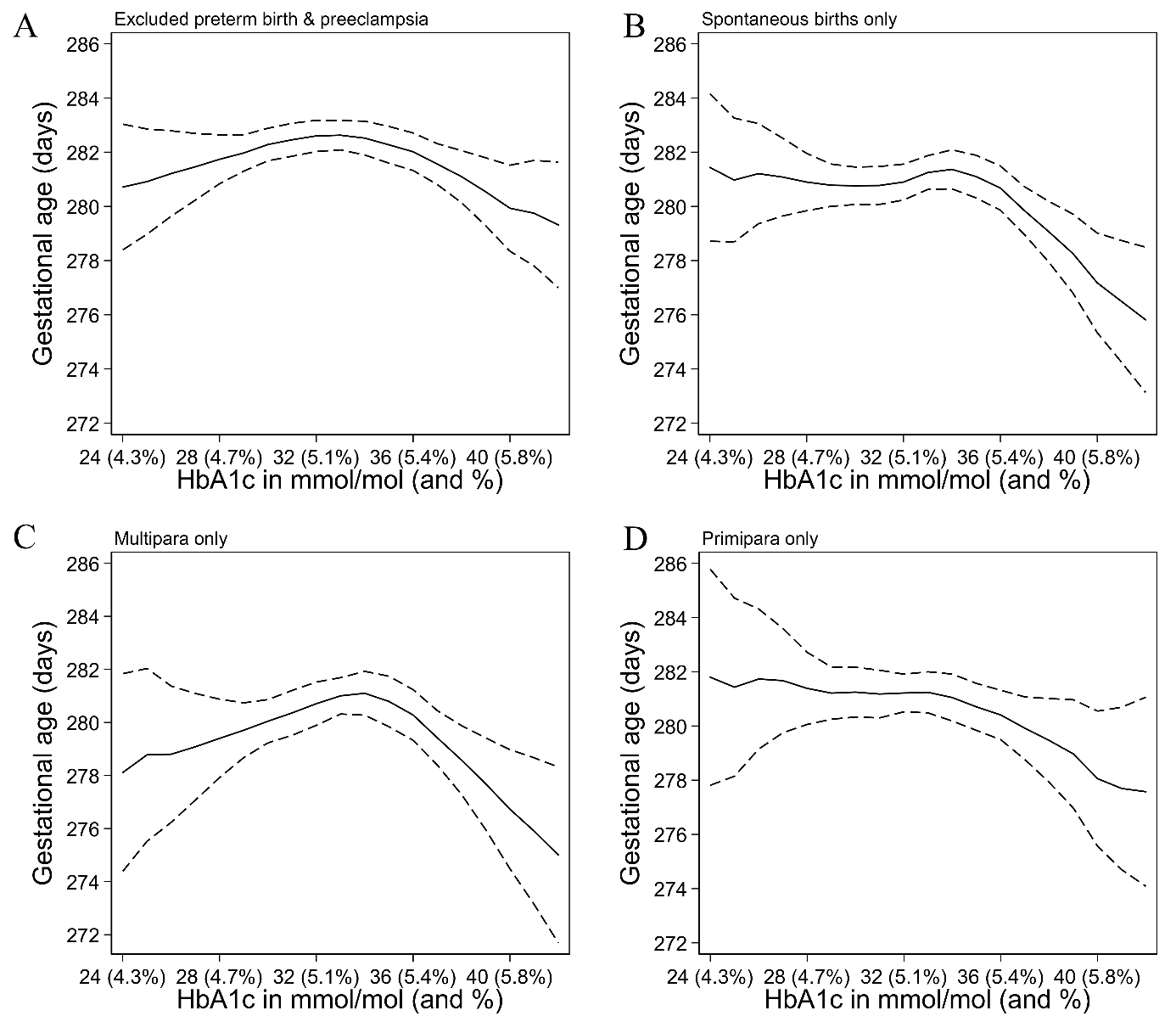


^a^ Removed n=87 preterm births and n=71 term births with preeclampsia.

^b^ The predictions were made using a linear regression model with restricted cubic splines for HbA1c with 4 knots (placed at 28, 31, 34 and 38 mmol/mol for panel A, B and 29, 32, 43 and 38 for panel C and 28, 31, 33 and 37 for panel D). Predictions were estimated using the adjusted models with the following covariates: maternal age (set to mean), body mass index (BMI) (set to mean), smoking (set to non-smokers), and parity (set to primipara, not included in panels C and D). Note that HbA1c levels were grouped at the extremes, with 42+ mmol/mol (6%) as the highest value (16 observations) and 24 mmol/mol (4.3%) as the lowest value (5 observations).

| **Supplementary Table S1:** Associations between different covariates and glycated haemoglobin (HbA1c) levels. Unadjusted associations with 95% confidence intervals and associated p-values, adjusted for maternal age and adjusted for maternal body mass index (BMI; kg/m^2^), and lastly adjusted for both maternal age and BMI. | | | | | | | | |
| --- | --- | --- | --- | --- | --- | --- | --- | --- |
| **Variable** | **Unadjusted model,** estimated change in HbA1c (95% confidence interval) | **p-value** | **Adjusted for maternal age,** estimated change in HbA1c (95% confidence interval) | **p-value** | **Adjusted for maternal BMI,** estimated change in HbA1c (95% confidence interval) | **p-value** | **Adjusted for maternal age and BMI,** estimated change in HbA1c (95% confidence interval) | **p-value** |
| **Maternal age** | 0.11 (0.09;0.14) | <0.001 | - |  | 0.11 (0.09;0.14) | <0.001 | - |  |
| **Maternal body mass index (BMI)** | 0.08 (0.05;0.11) | <0.001 | 0.08 (0.05;0.10) | <0.001 | - |  | - |  |
| **Parity** |  |  |  |  |  |  |  |  |
| Multipara vs primipara | 0.30 (0.09; 0.51) | 0.005 | -0.04 (-0.26;0.18) | 0.75 | 0.30 (0.09;0.51) | 0.004 | -0.3 (-0.25;0.19) | 0.82 |
| **Smoking at 18 weeks** |  |  |  |  |  |  |  |  |
| Smoker vs non-smoker | 0.56 (0.12;0.99) | 0.01 | 0.58 (0.15;1.01) | 0.009 | 0.52 (0.08;0.97) | 0.02 | 0.54 (0.11;0.97) | 0.02 |
| Smoker or quitter vs non-smoker | 0.17 (-0.10;0.44) | 0.22 | 0.23 (-0.04;0.50) | 0.09 | 0.16 (-0.11;0.43) | 0.26 | 0.22 (-0.05;0.48) | 0.11 |
| Quitter vs non-smoker | -0.03 (-0.35;0.29) | 0.86 | 0.04 (-0.27;0.35) | 0.80 | -0.03 (-0.35;0.28) | 0.83 | 0.04 (-0.28;0.35) | 0.81 |
| **Maternal education** |  |  |  |  |  |  |  |  |
| < High school | 0.13 (-0.39;0.65) | 0.63 | 0.28 (-0.23;0.79) | 0.29 | 0.07 (-0.45;0.60) | 0.79 | 0.21 (-0.31;0.73) | 0.42 |
| High school | 0.01 (-0.24;0.27) | 0.93 | 0.16 (-0.09;0.41) | 0.21 | -0.01 (-0.27;0.24) | 0.92 | 0.14 (-0.11;0.39) | 0.28 |
| College ≤ 4 years (reference) | 1 |  | 1 |  | 1 |  | 1 |  |
| >4 years college | 0.00 (-0.27;0.27) | 0.99 | -0.22 (-0.49;0.05) | 0.11 | 0.03 (-0.24;0.30) | 0.81 | -0.18 (-0.45;0.09) | 0.19 |
| **Native language** |  |  |  |  |  |  |  |  |
| Foreign-speaker vs Norwegian | 0.18 (-0.21;0.57) | 0.37 | 0.12 (-0.27;0.50) | 0.55 | 0.20 (-0.19;0.60) | 0.31 | 0.14 (-0.24;0.53) | 0.47 |
| **Sex** |  |  |  |  |  |  |  |  |
| Girl vs boy | -0.03 (-0.24;0.18) | 0.76 | -0.04 (-0.24;0.17) | 0.72 | -0.03 (-0.23;0.18) | 0.81 | -0.03 (-0.24;0.18) | 0.78 |
| **Weight gain before week 18** | -0.001 (-0.04;0.04) | 0.94 | -0.02 (-0.06;0.01) | 0.20 | 0.03 (-0.01;0.07) | 0.17 | 0.00 (-0.04;0.04) | 0.91 |
| **Maternal height** | 0.94 (-0.86;2.75) | 0.31 | 0.60 (-1.18;2.38) | 0.51 | 1.29 (-0.52;3.10) | 0.16 | 0.95 (-0.84;2.73) | 0.30 |
| **Use of assisted reproductive technologies (ART)** |  |  |  |  |  |  |  |  |
| ART versus non-ART | -0.07 (-0.82; 0.69) | 0.86 | -0.42 (-1.17; 0.33) | 0.28 | -0.08 (-0.84; 0.67) | 0.83 | -0.43 (-1.17; 0.32) | 0.26 |

**Supplementary Table S2**: Comparisons of model fits by likelihood ratio tests of regression analyses of glycated haemoglobin (HbA1c) measured at 18 gestational weeks and multiple perinatal outcomes. Singleton pregnancies, Norway, 2002-2009.

| **Outcome ^a^** | **P-value for likelihood-ratio-test of HbA1c as restricted cubic splines (4 knots) vs linear continuous** | **P-value for likelihood-ratio-test of HbA1c as restricted cubic splines (4 knots) vs linear spline 1 knot** |
| --- | --- | --- |
| **Birthweight Z-score** | 0.69 | NA ^b^ |
| **Length Z-score** | 0.43 | NA ^b^ |
| **Head circumference Z-score** | 0.70 | NA ^b^ |
| **Large-for-gestational age** | 0.99 | NA ^b^ |
| **Small-for-gestational age** | NA ^b^ | 0.67 |
| **Preterm birth** | NA ^b^ | 0.27 |
| **Preeclampsia** | NA ^b^ | 0.13 |
| **Congenital malformations** | NA ^b^ | 0.71 |
| **Gestational age** | NA ^b^ | 0.59 |

^a^ Adjusted for: maternal age (whole years), maternal pre-pregnancy body mass index (BMI) (kg/m^2^), smoking in pregnancy (yes versus no). Also adjusted for parity (0 versus 1+) for gestational age. Z-score and small and large-for-gestational-age standardized for gestational age in whole weeks, parity (0 vs 1+) and sex. Large-for-gestational age was defined as >90^th^ percentile, and small-for-gestational age as <10^th^ percentile.

^b^ NA: Not assessed

**Supplementary Table S3**: Linear single-knot spline regression analyses for glycated haemoglobin (HbA1c) measured at 18 gestational weeks and gestational age. Sensitivity analyses. Singleton pregnancies, Norway, 2002-2009.

| **Model for gestational age in days ^a^** | **HbA1c level ≤34 mmol/mol**, ^b^ estimate per unit (95% CI) | **HbA1c level ≥35 mmol/mol,** ^b^ estimate per unit (95% CI) | **Number of observations for each model**  no. (% of study sample) |
| --- | --- | --- | --- |
| **Excluded preterm births and pregnancies with preeclampsia** | 0.08 (-0.09 to 0.25) p=0.35 | -0.52 (-0.83 to -0.22) p=0.001 | 2719 (92.6) |
| **Term births only** | 0.08 (-0.08 to 0.25) p=0.32 | -0.53 (-0.83 to -0.24) p<0.001 | 2789 (95.0) |
| **Non-preeclamptic births only** | 0.02 (-0.17 to 0.21) p=0.86 | -0.65 (-0.99 to -0.30) p<0.001 | 2789 (95.0) |
| **Spontaneous onset of delivery only** | 0.08 (-0.12 to 0.27) p=0.43 | -0.71 (-1.06 to -0.35) p<0.001 | 2391 (81.4) |
| **Multipara only** | 0.26 (-0.02 to 0.53) p=0.07 | -0.79 (-1.24 to -0.34) p=0.001 | 1397 (47.6) |
| **Primipara only** | -0.11 (-0.38 to 0.16) p=0.43 | -0.54 (-1.07 to -0.02) p=0.04 | 1477 (50.3) |

^a^ Adjusted for: maternal age (whole years), maternal pre-pregnancy body mass index (BMI) (kg/m^2^), smoking in pregnancy (yes versus no). Also adjusted for parity (0 versus 1+) in the models excluding preterm births and preeclampsia and in the model only looking at spontaneous onset of delivery.

^b^ Showing the single-knot linear spline regression coefficients for the linear slope of HbA1c level up to and including 34 mmol/mol and from 35 mmol/mol and up, respectively.

References

1. Irgens LM. The Medical Birth Registry of Norway. Epidemiological research and surveillance throughout 30 years. Acta obstetricia et gynecologica Scandinavica 2000;79:435-9.

2. Magnus P, Birke C, Vejrup K, et al. Cohort Profile Update: The Norwegian Mother and Child Cohort Study (MoBa). International journal of epidemiology 2016;45:382-8.

3. Ronningen KS, Paltiel L, Meltzer HM, et al. The biobank of the Norwegian Mother and Child Cohort Study: a resource for the next 100 years. Eur J Epidemiol 2006;21:619-25.

4. Paltiel L, Haugan A, Skjerden T, et al. The biobank of the Norwegian Mother and Child Cohort Study – present status. Norsk Epidemiologi 2014;24.

5. Caspersen IH, Thomsen C, Haug LS, et al. Patterns and dietary determinants of essential and toxic elements in blood measured in mid-pregnancy: The Norwegian Environmental Biobank. Sci Total Environ 2019;671:299-308.

6. Selvin E, Coresh J, Jordahl J, Boland L, Steffes MW. Stability of haemoglobin A1c (HbA1c) measurements from frozen whole blood samples stored for over a decade. Diabet Med 2005;22:1726-30.

7. Rolandsson O, Marklund SL, Norberg M, Agren A, Hägg E. Hemoglobin A1c can be analyzed in blood kept frozen at -80 degrees C and is not commonly affected by hemolysis in the general population. Metabolism 2004;53:1496-9.

8. L. K. Validation of New Enzymatic Abbott Architect c8000 HbA1c Assay. Finland: Metropolia University of Applied Sciences; 2013.

9. National Glycohemoglobin Standardization Program, Factors that Interfere with HbA1c Test Results, 2019, ngsp.org/factors.asp (2 August 2020, date last accessed)

10. Church D, Simmons D. More evidence of the problems of using HbA1c for diagnosing diabetes? The known knowns, the known unknowns and the unknown unknowns. J Intern Med 2014;276:171-3.

11. Rafat D, Ahmad J. HbA1c in pregnancy. Diabetes Metab Syndr 2012;6:59-64.

12. Lilleholt K, Hallberg MH, Hagve TA. [Hemoglobinopathies and patients with foreign names]. Tidsskr Nor Laegeforen 2005;125:1164-7.

13. International Association of Diabetes Pregnancy Study Groups Consensus Panel, Metzger BE, Gabbe SG, et al. International association of diabetes and pregnancy study groups recommendations on the diagnosis and classification of hyperglycemia in pregnancy. Diabetes Care 2010;33:676-82.

14. World Health Organization. 2, Glycated haemoglobin (HbA1c) for the diagnosis of diabetes. Use of Glycated Haemoglobin (HbA1c) in the Diagnosis of Diabetes Mellitus: Abbreviated Report of a WHO Consultation. Geneva: World Health Organization, ; 2011.

15. American Diabetes Association. 2. Classification and Diagnosis of Diabetes: Standards of Medical Care in Diabetes-2020. Diabetes Care 2020;43:S14-s31.

16. Nielsen LR, Ekbom P, Damm P, et al. HbA1c levels are significantly lower in early and late pregnancy. Diabetes Care 2004;27:1200-1.
